# A prospective observational study of nurses performing minimally invasive tissue sampling of brain, liver, and lung tissues among deceased neonates and stillbirths in Ethiopia

**DOI:** 10.3389/fped.2023.1278104

**Published:** 2023-12-07

**Authors:** Lulu Mussa Muhe, Christina R. Paganelli, Rahell Hailu Ayele, Tigist Desta, Amha Mekasha, Asrat Demtse, Tesfamichael Awoke, Teferi Elfu, Tewodros Yalew Gebremariam, Dagnachew Tamrat, Amanuel Damie Jiffar, Aklilu Mekasha Zenabu, Moti Sori, Lindsay Parlberg, Alemayehu Worku, Assaye Kassie Nigussie

**Affiliations:** ^1^ College of Health Sciences, Addis Ababa University, Addis Ababa, Ethiopia; ^2^ Social, Statistical and Environmental Sciences, Research Triangle Institute (RTI) International, Durham, NC, United States; ^3^ Pathology Unit, Armauer Hansen Research Institute (AHRI), Addis Ababa, Ethiopia; ^4^ Neonatology Unit, Butajira General Hospital, Butajira, Ethiopia; ^5^ Paediatrics and Child Health Department, Bahir Dar University, Bahir Dar, Ethiopia

**Keywords:** task-shifting, neonatal mortality, MITS, stillbirths, community

## Abstract

**Background:**

With a neonatal mortality rate of 33 per 1,000 live births in 2019, Ethiopia is striving to attain the Sustainable Development Goal target of 12 deaths per 1,000 live births by 2030. A better understanding of the major causes of neonatal mortality is needed to effectively design and implement interventions to achieve this goal. Minimally Invasive Tissue Sampling (MITS), an alternative to conventional autopsy, requires fewer resources and through task-shifting of sample collection from pathologists to nurses, has the potential to support the expansion of pathology-based post-mortem examination and improve mortality data. This paper evaluates the accuracy and adequacy of MITS performed by nurses at a tertiary and general hospital and in the home of the deceased.

**Methods:**

Nurses in a tertiary and general hospital in Ethiopia were trained in MITS sample collection on neonatal deaths and stillbirths using standardized protocols. MITS sample collection was performed by both pathologists and nurses in the tertiary hospital and by nurses in the general hospital and home-setting. Agreement in the performance of MITS between pathologists and nurses was calculated for samples collected at the tertiary hospital. Samples collected by nurses in the general hospital and home-setting were evaluated for technical adequacy using preestablished criteria.

**Results:**

One hundred thirty-nine MITS were done: 125 in hospitals and 14 inside homes. There was a perfect or almost perfect agreement between the pathologists and the nurses in the tertiary hospital using Gwet's agreement interpretation criteria. The adequacy of MITS samples collected by nurses in the general hospital was more than 72% when compared to the preset criteria. The adequacy of the MITS sampling yield ranged from 87% to 91% on liveborn neonatal deaths and 76% for the liver, right and left lungs and 55% for brain tissues in stillbirths.

**Conclusions:**

This study demonstrated that task-shifting MITS sample collection to nurses can be achieved with comparable accuracy and adequacy as pathologists. Our study showed that with standardized training and supportive supervision MITS sample collection can be conducted by nurses in a tertiary, general hospital and, at the home of the deceased. Future studies should validate and expand on this work by evaluating task-shifting of MITS sample collection to nurses within community settings and with larger sample sizes.

## Introduction

Globally countries are striving to meet the Sustainable Development Goal (SDG) target of reducing neonatal mortality to less than 12 per 1,000 livebirths by 2030 (1). The neonatal mortality rate in Ethiopia in 2019 was 33 per 1,000 livebirths (2) and it has not decreased since 2017 (2). To attain the SDG target of reducing neonatal mortality to fewer than 12 neonatal deaths per 1,000 livebirths targeted interventions need to be scaled up.

An accurate understanding of cause of death is needed to identify and inform interventions to reduce neonatal deaths and stillbirths. This is especially true in resource-constrained settings such as Ethiopia, where the cause of death is rarely medically certified and even when causes are documented, they are often based on sources such as verbal autopsy, which has variable validity and accuracy (3–5). The gold standard for determining cause of death, the Complete Diagnostic Autopsy (CDA), is recognized to be the most accurate post-mortem examination method (6, 7). However, it is resource intensive, requires pathologists, and is time consuming (8). In 2019 it was estimated that Ethiopia had fewer than 100 pathologists nationwide, resulting in a ratio of one pathologist per one million population (9, 10), few of whom work in rural settings where most stillbirths, neonatal and young infant deaths occur. Minimally Invasive Tissue Sampling (MITS), a pathology-based post-mortem examination typically conducted by pathologists, consists of transcutaneous needle sampling of organs and body fluids for histological and microbiological analysis (11–13). MITS has been validated against CDA, including comparing CDA to MITS in neonates in a large tertiary hospital in Addis Ababa (14, 15), Ethiopia. Because MITS requires fewer resources, is less invasive, and is more acceptable to families (16, 17), the utility of MITS in resource-constrained settings is high and more so if MITS can be conducted by health workers with less specialized training than pathologists.

In 2020 the Ethiopia Nurses Association estimated there were more than 43,000 nurses nationwide (one nurse per 2,299 population), most of whom work at the primary care level (9, 10). The process of shifting specific tasks from highly qualified health workers to health workers with shorter training is called task-shifting. Task-shifting has been shown to increase efficiency, cost effectiveness and improve access within health systems (18–21). When implemented with standardized training and quality assurance processes, task-shifting MITS sample collection from pathologists to nurses has the potential to reduce barriers to improving mortality data for stillbirths and neonates in primary and community settings.

To date, the operational feasibility and effectiveness of task-shifting within the context of MITS has not been systematically evaluated. The objective of this study was to assess the accuracy and adequacy of MITS samples collected by nurses compared with pathologists in a tertiary hospital and to assess the operational feasibility of nurses performing MITS at a general hospital and in the home of the deceased.

## Methods

### Study design

This was an observational study using standardized procedures and protocols to prospectively collect MITS samples in a tertiary hospital, a general hospital and in the homes of deceased stillbirths and neonates. Prior to the initiation of MITS sample collection formative research, including community engagement involving community leaders, was conducted to inform study design and implementation.

### Settings

In March 2020 through February 2021 MITS samples were collected by both pathologists and nurses at Ethiopia's largest referral hospital, Tikur Anbessa Specialized Hospital (TASH) Neonatal Intensive Care Unit (NICU) in the capital city of Ethiopia, Addis Ababa. TASH has a well-organized 40-bed capacity NICU.

Beginning March 2021 through August 2022 MITS samples were collected by trained nurses working in Butajira General Hospital (BGH), in the town of Butajira, located 138 km south of Addis Ababa. BGH serves rural communities in Butajira District and is part of the Butajira Health and Demographic Surveillance Site (HDSS), established in 1987 (22). BGH has a delivery room and small 12-bed NICU. MITS samples were collected from neonatal deaths and stillbirths occurring both in the hospital as well as deaths occurring at home. Unlike at TASH, MITS sampling at BGH was not done by the pathologists as it was logistically infeasible. Instead, a team of pathologists reviewed the adequacy of the samples collected by the nurses using preset evaluation criteria.

### Training of nurses and pathologists

Nurses were trained to perform MITS using a standardized training curriculum (23). Five nurses from TASH and twelve nurses from BGH, were trained on MITS sample collection. These trainings were conducted by senior pathologists experienced in MITS. Training of the nurses consisted of a one-day mostly didactic session followed by 4 days of practical training collecting lung, liver, and brain tissues. Per the standardized curriculum, training facilitators utilized a step-by-step checklist to ensure that nurses performed MITS sample collection completely and consistently. Throughout the course of the study senior investigators and pathologists provided in-person and virtual supportive supervision to reinforce the nurses' skills and ensure sample quality.

## Inclusion and exclusion criteria

The inclusion criteria for MITS were neonatal deaths occurring in the hospital before the age of 28 days and stillbirths in both TASH and BGH. Inclusion criteria for BGH also included stillbirths born in the NICU or at home within the HDSS catchment area. In both settings the exclusion criteria included deaths where MITS could not be completed within 24 h of death or parents or legal guardians refused consent.

## Consent

Written consent for MITS was obtained from the parents or legal guardians in Amharic, the local language, within 24 h of death for both hospital and home deaths.

## Specimen collection

In both TASH and BGH, tissue samples were obtained from brain, lungs (left and right) and the liver per the MITS Surveillance Alliance standard operating procedures for MITS sample collection (24). Blood and CSF specimens were not collected as part of this study. A sample was considered adequate if a minimum of two cores of target tissues were collected for each organ. Target tissue for the brain was defined as a sample including grey matter, white matter, ependymal, meninges, and at least one intact (unfragmented) large tissue bit identified for brain. Target tissue for the lung was defined as a sample that included tissue from both small airways and large airways with at least one large and intact tissue segment. Liver target tissue was defined as including at least six portal tracts and at least one intact large tissue segment (Supplementary Material S1). In the TASH study, MITS was performed twice for each recruited death, first by a nurse followed by a pathologist, both trained in MITS sample collection; at BGH only nurses collected samples.

### Criteria for adequacy of tissue samples

A team of four pathologists independently assessed the quality of all samples to determine if adequate target tissue was obtained using preset histopathological criteria (25). A consensus of the histopathological findings is reached by the four pathologists to categorize the results as follows:

(1) Technically adequate if there were 2 or more full cores of target tissue or if there was significant tissue that was diagnostically relevant.
(2) Technically inadequate if there were <2 cores of the target tissue or no significant findings.
(3) No target tissue if the sample lacked target tissue and only contained diagnostically irrelevant tissue (e.g., skin or cartilage).

### Data collection and analysis

Structured clinical summary forms and standardized MITS sample collection forms were used to collect relevant clinical information, document the MITS procedure and the histopathological findings (Supplementary Material S2). Tissue specimens were fixed in 10% neutral buffered formalin for 24 h and stained with hematoxylin and eosin as per standard procedures. The samples were then evaluated histologically using light microscopy.

Data was initially collected on pretested case reporting forms and subsequently entered electronically on tablets. Data quality was monitored regularly. For samples from TASH, agreements on the identification of target tissue between pathologists and nurses were assessed using the first-order agreement coefficient, or the AC1 statistic which is proposed by Gwet (26). This was chosen over using Cohen's kappa due to the “kappa Paradox”, explained by Feinstein and Cicchetti (27), where a high value of observed agreement (*P*_o)_ is substantially lowered due to the imbalance in the contingency table marginal totals either vertically or horizontally. This appears to be the case in our sample where Kappa is negative despite a high level of agreement. Gwet's AC1 statistic provides a chance corrected agreement coefficient which is in line with the observed level of agreement (28, 29). We used Kappa etc. package of Stata 18 (Stata Corp. 2023. Stata Statistical Software: Release 18. College Station, TX: Stata Corp LLC) to estimate Gwet's AC1 agreement coefficient and the 95% CI.

Samples collected at BGH were evaluated calculating the proportions of technically adequate or diagnostically relevant samples compared with the proportion of inadequate samples or samples containing no target tissue. Statistical significance was tested using the Chi-squared test at *p* < 0.05.

## Results

A total 139 MITS were conducted: 48 at TASH, 77 at BGH and 14 inside homes of rural communities within Butajira district.

### Enrolment of neonatal deaths and stillbirths for MITS

During March 2020–February 2021, there were 2,527 NICU admissions and 356 NICU deaths eligible for MITS at TASH. A total of 170 families were approached, and consent was obtained for 50 (29.4%) deaths. Two cases were excluded because death occurred after the age of 28 days i.e., outside the neonatal period. Samples from 48 MITS cases were included in the final analysis.

**Figure 1:**
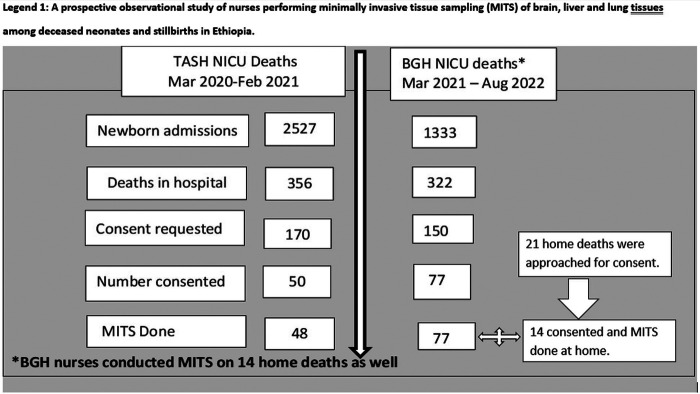
Flow diagram of enrolment.

Between March 2021 and August 2022 there were a total of 1,333 admissions and 322 deaths in BGH NICU, 150 of which were eligible, and approached for consent and 77 (51.3%) consented to MITS. Overall, consent rate was 39.7%. Due to local norms around reporting of perinatal deaths, the total number of home deaths in Butajira district during the study period could not be precisely determined; of the 18 deaths reported within the HDSS, 14 were eligible, approached and consented for MITS. Together with the home MITS, a total of 91 MITS was conducted by BGH nurses (Figure 1: Flow Diagram of Enrolment).

### Baseline characteristics of enrolled neonatal deaths and stillbirths

As shown in Table 1, a total of 139 MITS (68 males and 71 females) were conducted. There were 44 stillbirths, 36 of which occurred in hospital. Most liveborn deaths (77/94) happened within 7 days of birth. All cases had birthweight taken within 24 h of birth. Gestational age was not possible to estimate for most cases. Most neonatal deaths had a birthweight of 2,000 g or more; nine cases were extremely low birthweight (two in TASH and seven in BGH).

**Table 1:** Demographic characteristics of enrolled neonatal deaths and stillbirths.

	TASH *N* (%)	BGH *N* (%)	Total *N* (%)
Total enrolled	48 (35%)	91 (65%)	139
Sex			
Male	22 (46%)	46 (51%)	68 (49%)
Liveborn deaths	48	53	101
In hospital	48 (100%)	47 (90%)	95 (94%)
At home	0	6 (10%)	6 (6%)
Stillbirths	0	44	44
In hospital	0	36 (82%)	36 (82%)
At home	0	8 (18%)	8 (18%)

### Results of comparison of sample adequacy between nurses and pathologists at TASH

There was minimal or no autolysis of the samples. Both nurses and pathologists were able to perform MITS on 48 neonatal deaths and no stillbirths at TASH. Table 2 shows the number of adequate samples collected by nurses compared to pathologists. There was perfect agreement or almost perfect agreement (91.8%–100%) in terms of adequacy between samples collected by nurses and pathologists for all tissue types.

**Table 2:** Agreement in obtaining target tissue between pathologists and TASH nurses (*N* = 48).

Variables	Lt lung *N* = 48	Rt lung *N* = 48	Liver *N* = 48	Brain *N* = 48
Both pathologists and nurses obtained target tissue	45	44	48	46
Both missed target tissue	0	0	0	0
Only Pathologist missed target tissue	2	2	0	1
Only nurse missed target tissue	1	2	0	1
Agreement	93.9%	91.8%	100%	95.9%
Gwet AC1 (95% CI)^a^	0.93 (0.85–1.00)	0.91 (0.81–1.00)	1	0.96 (0.89–1.00)

^a^ Gwet's AC1 values interpretation is 0.01–0.20 slight agreement; 0.21–0.40 fair agreement; 0.41–0.60 moderate agreement; 0.61–0.80 substantial agreement; 0.81–1.00 almost perfect or perfect agreement (26).

### Results of sample adequacy at BGH hospital

MITS samples collected by BGH nurses were evaluated using preset criteria by an independent team of pathologists. As shown in Table 3, the adequacy of MITS samples in both liveborn deaths and stillbirths combined was 72%, 78%, 82% and 79% for the brain, liver, right lung and left lung tissue samples respectively. Sample adequacy was higher in liveborn deaths at 91%, 87%, 91% and 87% for brain, liver, right lung and left lung samples respectively; compared to stillbirths with 55% sample adequacy for brain and 76% sample adequacy each for liver, right lung, and left lung samples. The difference in sample adequacy of brain tissue between neonates and stillbirths (91% vs. 55%) was significant (*p* < 0.05).

**Table 3:** Proportions of adequate samples of tissue collected by BGH nurses in hospital and home (*N* = 91).

Categories	Brain *N* = 88	Liver *N* = 91	Rt lung *N* = 91	Lt lung *N* = 91
Technically adequate (all)	63/88 (72%)	71/91 (78%)	75/91 (82%)	72/91 (79%)
Technically inadequate (all)	13/88 (15%)	9/91 (10%)	8/91 (9%)	7/91 (8%)
Liveborn deaths				
Technically adequate	41/45 (91%)^a^	39/45 (87%)	42/46 (91%)	40/46 (87%)
Technically inadequate	7 (16%)	6 (13%)	3 (7%)	3 (7%)
No target tissue	1 (2%)	1 (2%)	1 (2%)	3 (7%)
Stillbirths				
Technically adequate	22/40 (55%)^a^	32/42 (76%)	33/42 (76%)	32/42 (76%)
Technically inadequate	6 (15%)	3 (7%))	5 (12%)	4 (10%
No target tissue	8 (20%)	6 (14%)	4 (10%)	6 (14%)
Misplaced/mislabelled	3 (3%)	4 (4%)	3 (3%)	3(3%)

^a^ Chi-square testing comparing proportions of adequate vs. inadequate tissue vs. no target tissue for liveborn deaths vs. stillbirths was not significant except for brain samples where *X*^2 ^= −3.789 with *p*-value < 0.05.

Overall, the proportions of samples evaluated as either technically inadequate or missing target tissue was higher in stillbirths compared to neonates, 25% vs. 13.7%, respectively. The proportion of neonatal samples evaluated as technically inadequate or missing target tissue were highest in brain tissue (17.7%), followed by liver (15.5%), left lung (13%) and right lung (8.6%). Similarly, the proportion of stillbirth samples that were inadequate or lacking target tissue were highest in brain tissue (35%), followed by left lung (24.8%) and liver and right lung (21.4% each).

### Results of sample adequacy of samples collected in the home

Table 4 shows the proportions of adequate samples of tissue collected by nurses at home only. Of the 14 MITS conducted in the home, technically adequate tissue was obtained in 79%, 86% and 93% of the liver, the left lung, and the right lung tissue samples respectively. Only 57% of brain samples were evaluated at technically adequate. The sample size was too small to calculate statistical significance. There was no target tissue in three samples for the brain.

**Table 4:** Proportions of adequate samples of tissue collected by nurses at home only (*N* = 14).

Categories	Brain	Liver	Rt lung	Lt lung
Technically adequate	8 (57%)	11 (79%)	13 (93%)	12 (86%)
Technically inadequate	3 (21%)	1 (6%)	0	1 (6)
No target tissue	3 (21%)	2 (14%)	1 (7%)	1 (7%)
Total	14	14	14	14

### Nurse perceptions and experiences

On average, it took nurses 30.3 min to complete each MITS case including sample collection and preparation of samples for storage and transport. In contrast, on average, it took pathologists 25.2 min to complete each MITS. In addition to MITS sample collection, the nurses participated in community engagement activities for MITS, counselling families for consent, and notifying families of MITS results. Anecdotally nurses felt that families were more inclined to consent to MITS if their neonates were cared for in the NICU prior to death. Barriers to conducting MITS identified by the nurses included families' lack of awareness of MITS, family members' discomfort with the sound made by the sample collection instrument and technical challenges with electronic data entry. Overall nurses found the experience to be positive and all nurses stated that they would be supportive of broader implementation of MITS.

## Discussion

This is the first time a study has evaluated the feasibility and results of task-shifting MITS sample collection from pathologists to nurses. Our study showed that with standardized training and supportive supervision MITS sample collection can be conducted by nurses in a tertiary care setting, a general hospital and in the community, at the home of the deceased.

The adequacy of MITS samples collected by nurses was comparable to that of pathologists in the tertiary hospital, with perfect or almost perfect agreement. Overall, the proportion of adequate samples collected by nurses across all tissue types ranged from 91% to 100% in neonates for nurses at TASH and 87%–91% in neonates and 55%–76% in stillbirths for nurses at BGH. Observing the slightly higher proportions of adequate samples at TASH likely reflects a higher volume of MITS cases and increased opportunities for nurses to practice and hone their skills compared to BGH. Our study results showed obtaining adequate samples of brain tissue, particularly in stillbirths, was the most challenging samples for nurses to collect. While brain tissue was included as part of our sampling protocol, recent studies indicate that histological analysis of brain tissue has limited utility in determining cause of death in stillbirths. It is reasonable to reconsider the inclusion of brain tissue as part of MITS sampling in stillborn populations as part of future studies (30).

Human resource shortages in the health services are widely acknowledged as a threat to the attainment of the SDGs (1) and optimization of the existing health workforce is crucial to attain the SDGs. Redistribution of tasks and responsibilities among cadres of health workers is seen as a promising strategy for improving access and cost effectiveness within health systems. Such task-shifting or sharing strategies might be particularly attractive to countries that lack the means to improve access to care in maternal and child health, in management of malaria, tuberculosis, HIV and other conditions within short periods of time (31–33). Task shifting to nurses and midwives has been demonstrated as an effective strategy to increase access to care without compromising outcomes and reduce costs across several health issues including reducing HIV transmission through male medical circumcision (34), supporting early infant diagnosis of HIV and expanding family planning through tubal ligation (35), performing point of care HIV testing (36), and obstetric surgery (37). Key components of effective and sustainable task-shifting programs include conducting high-quality standardized training, providing ongoing supervision, utilizing quality assurance processes, ensuring potential career progression and offering incentive packages for health workers. In addition, programs need to address the distribution of roles among cadres, regulatory issues, stakeholder involvement, systems for referral, and supply chains when shifting tasks from one health worker cadre to another (38). While we were not able to implement the broader questions such as regulatory issues at this stage, our study ensured that training and supervision, supplies and incentives were fully implemented.

Our study demonstrates that task-shifting MITS sample collection to nurses is a feasible strategy to increase the use of pathology-based post-mortem examination, improve mortality data and ultimately, reduce stillbirths and neonatal mortality.

## Limitations

Our study had several limitations. This was a small study comparing two cadres of health professions; a randomized controlled design and a larger sample size might have provided definitive results. The total number of MITS cases performed by nurses was limited making it difficult to determine how additional practice and oversight would have impacted the adequacy of samples. This study included a limited number of histology types (lung, liver and brain). Including microbiology and additional samples such as blood and cerebral spinal likely would have impacted the results.

## Conclusion

Our study showed that, except for brain tissue in stillbirths, nurses can conduct MITS sample collection with comparable accuracy and adequacy as pathologists. Task-shifting MITS sample collection was perceived as acceptable by the nurses participating in our study, noting that they felt competent and comfortable collecting MITS samples. We demonstrated the feasibility of conducting MITS both in a tertiary hospital, a general hospital and in the homes of the deceased. Task-shifting MITS sample collection to nurses and collecting MITS samples in community settings is an important step in paving the way for scaling-up of MITS sample collection both within and outside of health facilities. We recommend further validation of the performance of nurses as part of task-shifting of MITS on a larger scale involving both liveborn deaths and stillbirths at community levels.

## Data Availability

The original contributions presented in the study are included in the article/**Supplementary Material**, further inquiries can be directed to the corresponding author.
