# Supplementary figures and images for: A prospective observational study of nurses performing minimally invasive tissue sampling of brain, liver, and lung tissues among deceased neonates and stillbirths in Ethiopia
Source: Front Pediatr. 2023 Dec 7;11:1278104. doi: 10.3389/fped.2023.1278104 (PMC10740176; doi:10.3389/fped.2023.1278104)

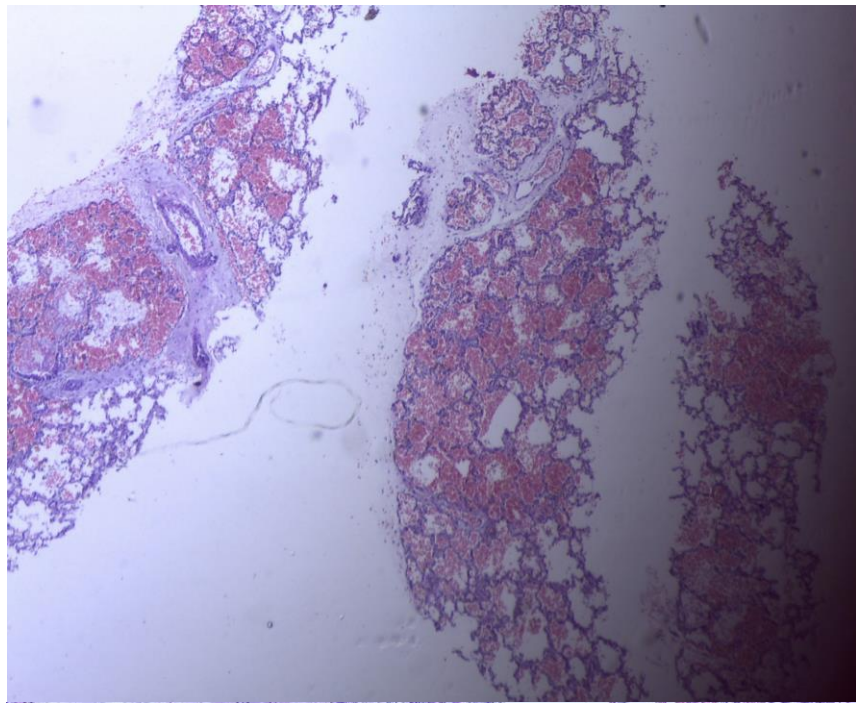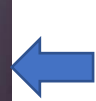

Lt lung

Liver

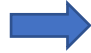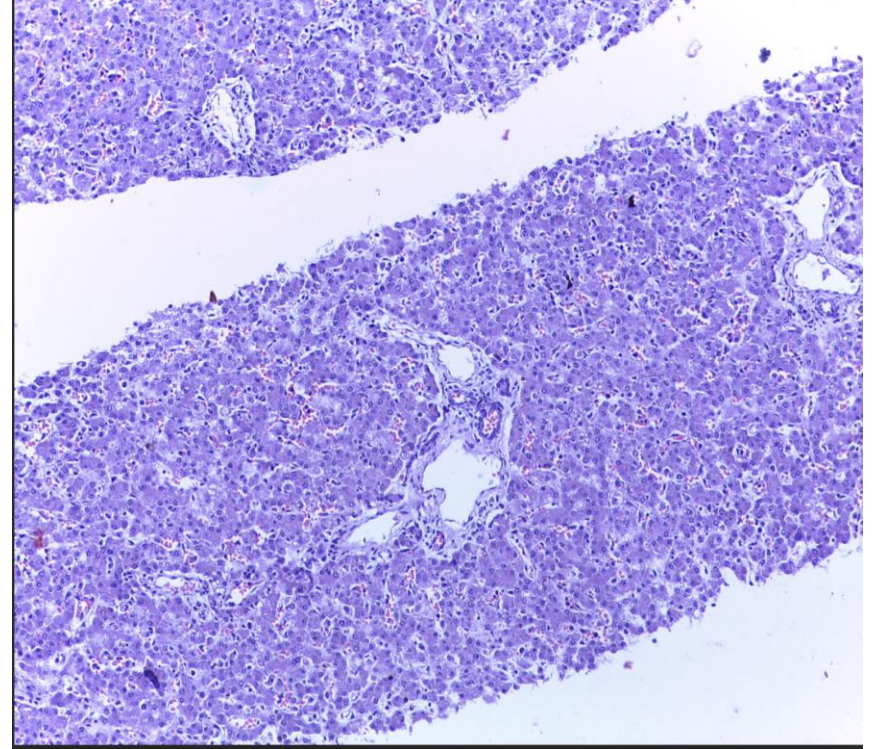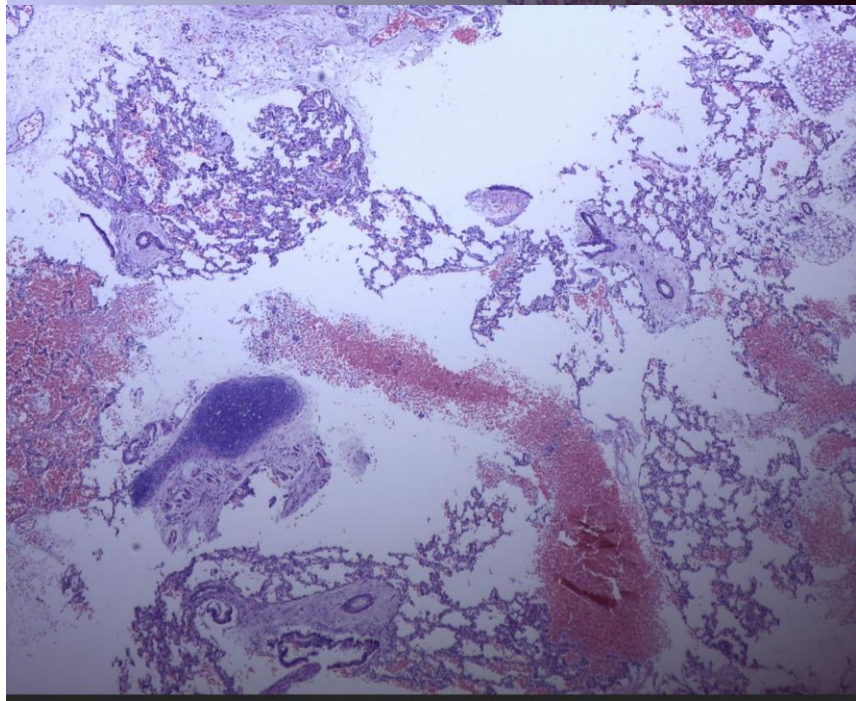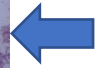

Rt lung

Brain

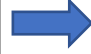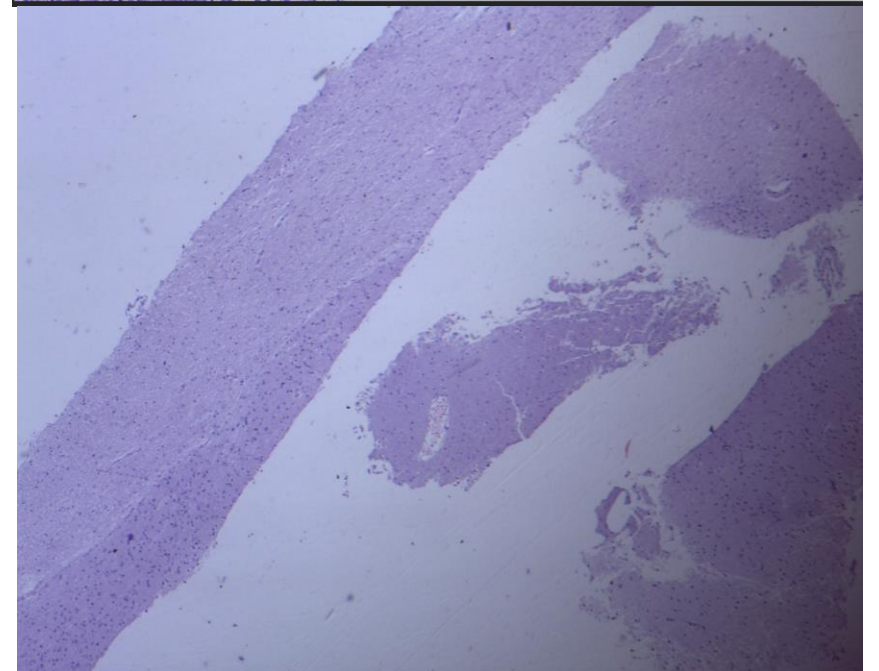

Supplement: Supplementary file 1 [file Datasheet1.pdf]
