# Supplementary material for: A prospective observational study of nurses performing minimally invasive tissue sampling of brain, liver, and lung tissues among deceased neonates and stillbirths in Ethiopia
Source: Front Pediatr. 2023 Dec 7;11:1278104. doi: 10.3389/fped.2023.1278104 (PMC10740176; doi:10.3389/fped.2023.1278104)
Supplement: Supplementary file 2 [file Datasheet2.pdf]

OBSTETRIC FORM		MSA_##
MITS SURVEILLANCE ALLIANCE	Study ID: |___|___|___|___|___|___|___|___|___|___|	Version 1.0<br>Page **1 of 2**

The obstetric form should be completed by the study nurse following delivery for all women participating in the study whether the infant is stillborn or live born.

SECTION A. SOCIODEMOGRAPHIC DATA

1. Maternal age: |__|__| years
2. Education of mother
   - 1☐ NO formal schooling, illiterate 2☐ School →a. Years of schooling: |__|__|
   - 3☐ NO formal schooling, literate 4☐ DK
3. Occupation of mother
   - 1☐ Housewife 2☐ Government/Company 3☐ Self-employed
   - 4☐ Farmer 5☐ DK 6☐ Other, specify:____________________
4. Is this mother's first pregnancy? 1☐ YES 2☐ NO 3☐ DK

SECTION B. PAST PREGNANCY HISTORY – ***SKIP TO SECTION C IF FIRST PREGNANCY***

Note: If the response in this section is None, please code 00.

1. Prior abortions: |__|__| (pregnancies ending at <20 weeks, including medical termination and miscarriage)
2. Prior stillbirths: |__|__| (pregnancies ending at ≥20 weeks)
3. Prior Pregnancies with live births: |__|__|
4. Date of last delivery: |__|__|-|__|__|-|__|__|__|__| (DD-MM-YYYY)
   - a. If unknown, estimate how many months since last delivery |__|__|__|

SECTION C. CURRENT PREGNANCY

1. Antenatal care (ANC) received? 1☐ YES 2☐ NO 3☐ DK
   - a. If YES, Number of visits (whether in this or other hospital/clinics): |__|__|
2. Last Hemoglobin (Hgb) level during this pregnancy: |__|__|. |__|g/dL 1☐ ND/DK
3. Last HCT level during this pregnancy: |__|__|. |__| % 1☐ ND/DK

4.	Rh factor	1☐ Positive	2☐ Negative	3☐ ND/DK
5.	VDRL /Syphilis test	1☐ Reactive	2☐ Nonreactive	3☐ ND/DK
6.	HIV/AIDS serostatus	1☐ Positive	2☐ Negative	3☐ ND/DK
7.	Active tuberculosis	1☐ Positive	2☐ Negative	3☐ ND/DK
8.	Urine protein	1☐ Positive	2☐ Negative	3☐ ND/DK
9.	Urine culture for infection	1☐ Positive	2☐ Negative	3☐ ND/DK
10.	Malaria test in this pregnancy	1☐ Positive	2☐ Negative	3☐ ND/DK
11.	Hepatitis B	1☐ Positive	2☐ Negative	3☐ ND/DK
12.	Hepatitis C	1☐ Positive	2☐ Negative	3☐ ND/DK
13.	TT vaccination	1☐ YES	2☐ NO	3☐ DK

- a. If YES -> Indicate number of doses: 1☐ 1 dose
  - 2☐ 2 doses
  - 3☐ Other, specify: _________________
- b. If YES -> Indicate timing 1☐ Current pregnancy 2☐ Pre-pregnancy 3☐ DK

SECTION D. MATERNAL MEDICAL AND OBSTETRIC DISORDERS

1. Cardiac disease? 1☐ YES 2☐ NO 3☐ DK
2. Diabetes mellitus in current pregnancy? 1☐ YES 2☐ NO 3☐ DK
   - a. If YES type - > 1☐ Pre-gestational 2☐ Gestational 3☐ DK
3. Thyroid disease? 1☐ YES 2☐ NO 3☐ DK
   - a. If YES type - > 1☐ Hyperthyroidism 2☐ Hypothyroidism 3☐ DK
4. History of tuberculosis (clinical diagnosis) 1☐ YES 2☐ NO 3☐ DK
5. Anemia? 1☐ YES 2☐ NO 3☐ DK
6. Other disorders? 1☐ YES 2☐ NO 3☐ DK
   - a. If YES → Specify____
7. Any hypertensive disorders? 1☐ YES 2☐ NO 3☐ DK
   - a. If YES→ Specify type:
     - 1☐ Pre-eclampsia 2☐ Eclampsia
     - 3☐ Superimposed pre-eclampsia 4☐ Chronic hypertension
     - 5☐ Gestational Hypertension 6☐ DK
8. Antepartum hemorrhage (APH): 1☐ YES 2☐ NO 3☐ DK
   - a. If YES→ Specify
     - 1☐ Placenta previa 2☐ Abruptio placenta 4☐ DK
     - 3☐ Other Hemorrhage, specify______________________________
9. Clinical Chorioamnionitis: 1☐ YES 2☐ NO 3☐ DK

SECTION E. INTRAPARTUM AND IMMEDIATE POSTPARTUM

1. Spontaneous ROM: 1☐ YES 2☐ NO 3☐ DK
2. Induction of labor: 1☐ YES 2☐ NO 3☐ DK
   - a. If YES→ Specify primary indication:
     - 1☐ Fetal distress 2☐ IUGR/SGA
     - 3☐ Known stillbirth 4☐ Pre-eclampsia/eclampsia
     - 5☐ Macrosomia 6☐ Congenital anomalies
     - 7☐ APH 8☐ Oligohydramnios
     - 9☐ PROM 10☐ Other, specify____________________
3. First/only baby: Presentation:
   - 1☐ Vertex 2☐ Breech 3☐ Transverse 4☐ DK
   - 5☐ Other, specify: ____

ND= Not Done DK = Don't know

4. First/only baby: Delivery mode:
1☐SVD
2☐Assisted/Instrumental
3☐C-Section → a. Specify primary indication:
1☐Fetal distress 2☐IUGR/SGA
3☐Pre-eclampsia/eclampsia 4☐Cord prolapsed
5☐Prior Cesarean-Section 6☐Macrosomia
7☐Breech 8☐Multiple pregnancy
9☐Prolonged or obstructed labor 10☐Elective Cesarean-Section
11☐Stillbirth 12☐Indication unknown 13☐ Chorioamnionitis
14☐Other, specify: ____
4☐Delivery mode unknown
5☐Other, specify: ____

5. Is pregnancy:
1☐Singleton 2☐Twins (*complete PU02A*)
3☐Triplet (*complete PU02A*) 4☐4 or more babies (*complete PU02A*)

6. Was first/only baby FHR detected?
1☐ YES → a. Was a FHR monitored?
1☐YES 2☐NO 3☐DK
2☐ NO (None detected)
3☐ DK

7. Date and time of delivery first/only baby:
a. Date: |__|__|-|__|__|-|__|__|__|__|(DD-MM-YYYY)

b. Time: |__|__| - |__|__|(HH) - (MM)

If multiples, please record the date and time of delivery for each baby. For singletons, please leave blank: **Second baby:**

c. Date: |__|__|-|__|__|-|__|__|__|__|(DD-MM-YYYY)
d. Time: |__|__| - |__|__|(HH) - (MM)

If multiples, please record the date and time of delivery for each baby. For singletons, please leave blank: **Third baby:**

e. Date: |__|__|-|__|__|-|__|__|__|__|(DD-MM-YYYY)
f. Time: |__|__| - |__|__|(HH) - (MM)

8. If labor, overall duration: |__|__|HH |__|__|MM 1☐ NA

9. Time of rupture of membrane: |__|__| - |__|__|(HH) - (MM) 1☐ NA
(Record '00, 00' if at delivery)

10. Delivery attendant *(Indicate most senior care giver at the delivery)*:
1☐ Obstetrician 2☐ Non-OB 3☐ Nurse/nurse midwife
4☐ Traditional Birth Attendant 5☐ Family (NO health provider)
6☐ Self-delivery 7☐ DK 8☐ Other, specify: ____________________

SECTION F. COMPLICATIONS DURING LABOR AND DELIVERY

1. Maternal fever prior to delivery: 1☐ YES 2☐ NO 3☐ DK
2. First/only baby; Cord prolapse: 1☐ YES 2☐ NO 3☐ DK
3. Ruptured Uterus: 1☐ YES 2☐ NO 3☐ DK
4. Other, specify: ____

SECTION G. MATERNAL MEDICATIONS PRIOR TO DELIVERY

1. Antibiotics 1☐ YES 2☐ NO 3☐ DK
a. If YES, **check all** indications for which antibiotics are given:
a☐ PROM b☐ Chorioamnionitis c☐ Urinary tract infection
d☐ Prophylaxis e☐ DK f☐ Other, specify: ___________

2. Steroids for fetal lung maturation 1☐ YES 2☐ NO 3☐ DK
a. If YES, Number of doses: |__| 1☐ DK
b. If YES, Time from the last dose of steroid to delivery:
|__|__|Days|__|__| HH

3. Magnesium sulfate: 1☐ YES 2☐ NO 3☐ DK
4. Diazepam: 1☐ YES 2☐ NO 3☐ DK
5. Tocolytics 1☐ YES 2☐ NO 3☐ DK
6. Progesterone: 1☐ YES 2☐ NO 3☐ DK
7. Pethidine: 1☐ YES 2☐ NO 3☐ DK
8. Antihypertensive 1☐ YES 2☐ NO 3☐ DK
9. Transfusion given 1☐ YES 2☐ NO 3☐ DK
10. Other medication: 1☐ YES 2☐ NO 3☐ DK
a. If YES, Other, Specify:
____

SECTION H. SAMPLE COLLECTION FOR ALL PRETERMS AND STILLBIRTHS – FIRST/ONLY BABY

1. First/only baby; Was cord blood sample collected? 1☐YES 2☐NO 3☐DK
2. First/only baby; Was placenta sample collected? 1☐YES 2☐NO 3☐DK

SECTION J. COMPLETION OF FORM

1. ID of person completing this form: |___|___|___|___|
a. Date form completed: |__|__|-|__|__|-|__|__|__|__| (DD-MM-YYYY)
2. ID of person reviewing this form: |___|___|___|___|
a. Date reviewed: |__|__|-|__|__|-|__|__|__|__| (DD-MM-YYYY)
3. ID of data entry person: |___|___|___|___|
a. Date of data entry: |__|__|-|__|__|-|__|__|__|__| (DD-MM-YYYY)

ETHIOPIA TASK SHIFTING STUDY ON MITS		
HOSPITAL NAME________	**CLINICAL SUMMARY FORM**	
	Study ID: |___|___ |___|___|___|___|___| MEDICAL RECORD:______________________________<br>Name:____	

This MITS study examination should be completed for the infant who died and consent is obtainedU.

SECTION 1. IDENTIFICATION

1. Date of birth: |__|__|-|__|__|-|__|__|__|__| (DD – MM – YYYY)
2. Time of birth (time in 12 hours/min): |__|__|-|__|__|(HH-MM) Check: 1☐AM 2☐PM 2☐DK
3. Infant Sex 1 ☐ Female 2 ☐ Male
4. Birth weight (in grams): |__|__|__|__| g or ☐Don't Know (Check if unknown or if taken >24 hrs)
5. Birth place: 1 ☐ Hospital (Name____________________________________)
   2 ☐ Health Center (Name____________________________________)
   3 ☐ Home 4 ☐ Other; specify:_________________

Home address:_________________________ Gott/Ketana__________________________
Zone:________________________________ Town or Woreda:_______________________________
Kabele:____
Phone: ____
Health Extension Worker Name:_______________________ (if known, cell number):________________
Description of location of residence (other detail as needed):

6. Best gestational age in weeks and days: |__|__| wks |__| days

SECTION 2. HISTORY OF INFANT'S SYMPTOMS SEEN BY PARENTS OR CAREGIVER PRIOR TO THE NICU ADMISSION. Complete this section for babies who arrive at the NICU from home, transferred from another facility. Check "Yes" "No" or "Don't Know/Not applicable" for each.

7. Was breathing normal?	1☐Yes	2☐No	3☐Don't know
8.1 If abnormal, tick all apply:	1☐Fast breathing<br>3☐Grunting<br>5☐Cyanosis	2☐Periods of apnea/not breathing at all<br>4☐Gasping (difficulty breathing)<br>6☐Don't know	
8. Was infant able to feed?	1☐Yes	2 ☐No	3☐Don't know
9. Was infant vomiting?	1☐Yes	2 ☐No	3☐Don't know
10.1 If vomiting, check type:	*1*☐Ingested milk	2☐Dark or coffee ground material	3☐Bilious or yellow – green colored
10. Did infant have normal temperature?	1☐Yes	2 ☐No	3☐Don't know
11.1 If no, specify		1☐Fever	2☐Cold to touch

11. Were body movements normal?	1☐Yes	2 ☐No		3☐Don't know
12. Was baby's color normal?	1☐Yes	2 ☐No		3☐Don't know
13. Was the abdomen normal?	1☐Yes	2 ☐No		3☐Don't know
14. Was the umbilicus normal?	1☐Yes	2 ☐No		3☐Don't know
15.1 If no, was there bleeding?		1☐Yes	2☐No	3☐Don't know
15.2 If no, pus, discharge or excessive secretions?		1☐Yes	2 ☐No	3☐Don't know
15.3 If no, redness of skin around the umbilicus?		1☐Yes	2 ☐No	3☐Don't know

SECTION 3. VITAL SIGNS AND ANTHROPOMETRIC MEASUREMENTS AT NICU ADMISSION

16. Date of exam: |__|__|-|__|__|-|__|__|__|__| (DD – MM – YYYY)

16.1 Time of exam: |__|__|-|__|__|(HH-MM) 1☐AM 2☐PM

17.Postnatal age at exam (in hours if less than 72 hours or else record in days)

17.1 |__|__| hrs **OR** 17.2 |__|__| days |__|__| min

18. Respiratory rate __ __ __ breaths/minute 1☐Not done
19. Apical heart rate __ __ __ beats/minute 1☐Not done
20. Temperature __ __ .__ °Celsius 1☐Not done
21. Weight at exam: __ __ __ __ grams 1☐Not done
22. Length __ __ .__ cm 1☐Not done
23. Head circumference__ __ .__ cm 1☐Not done
24. O_2 Saturation __ __ % 1☐Not done

24.1 If O_2 sat done, was baby on oxygen? 1☐Yes 2☐No

25. Capillary refill ___ ___ sec 1☐Not done

Describe subsequent changes on vital signs below until death

SECTION 4. GENERAL APPEARANCE. IN THIS SECTION RECORD OBSERVATIONS BY DOCTOR OR NURSE.

27. Level of consciousness	1☐Alert	2☐Sleepy	3☐Comatose
28. Respiratory distress	1☐Yes	2☐No	3☐Don't know
29. Movement or activity	1☐Active	2☐Decreased	3☐Absent
30. Dysmorphic features	1☐Normal	2☐Dysmorphic	3☐Don't know
30.1 If dysmorphic, describe:	1☐Trisomy 13	2☐Trisomy 18	3☐Trisomy 21

	4☐Cleft lip	5☐Cleft palate	6☐Don't know
	2☐Abnormal (odd) facies	3☐Mandibular hypoplasia	7☐Other, specify: ___________________
31. Normal posture	1☐Yes	2☐No	3☐Don't know
32. Skin color. Tick all that apply.	1☐Pink	2☐Plethoric	3☐Pale
	4☐Cyanotic	5☐Jaundice	6☐Dusky
33. Skull normal	1☐Yes	2 ☐No	3☐Don't know
34. Eyes normal	1☐Yes	2 ☐No	3☐Don't know
35. Nose patent	1☐Yes	2☐No	3☐Don't know
36. Ears normal (shape, size/position and patency)	1☐Yes	2 ☐No	3☐Don't know
37. Mouth normal	1☐ Yes	2 ☐No	3☐Don't know
38. Neck normal	1☐Yes	2☐No	3☐Don't know
**SECTION 6. RESPIRATORY SYSTEM**			
39. Breathing normal	1☐Yes	2☐No	3☐Don't know
39.1 if no, tick all present:		1☐Chest indrawing<br>2☐Chest retractions<br>3☐Nasal flaring<br>4☐Grunting<br>5☐Unilateral crepitations<br>6☐Bilateral crepitations<br>7☐Unilateral decreased air entry<br>8☐Bilateral decreased air entry<br>9☐Periodic apnea<br>10☐Other; specify:________	
**SECTION 7. CARDIOVASCULAR SYSTEM**			
40. Heart exam normal	1☐ Yes	2☐No	3☐Don't know

40.1 If no, specify:		1☐Tachycardia<br>2☐Accentuated P2<br>3☐Gallop rhythm<br>4☐ Murmur<br>5☐ Other. Specify:_________	
**SECTION 8. GASTROINTESTINAL SYSTEM**			
41. Abdomen normal	1☐Yes	2 ☐No	3☐Don't know
41.1. If no, tick all that apply:		1☐Scaphoid<br>2☐Distended<br>3☐Defects (omphalocele, gastroschisis/exstrophy of bladder)<br>4☐Other. Specify:______________________	
42. Bowel sounds	1☐Normal	2☐Decreased	3☐Absent 4☐Hyperactive
43. Organ size normal	1☐Yes	2 ☐No	3☐Don't know
43.1 If no, specify:	1☐Hepatomegaly	2☐Splenomegaly	3☐Abdominal mass
44. Anus patent	1☐Yes	2☐No	3☐Don't know
45. Stool present	1☐Yes	2☐No	3☐Don't know
45.1 If yes, specify:	1☐Normal/yellow	2☐Meconium	3☐Bloody
**SECTION 9. GENITOURINARY SYSTEM**			
46. Urine output normal	1☐Yes	2☐No	3☐Don't know
47. Genitalia/kidneys normal	1☐Yes	2☐No	3☐Don't know
47.1 If no, specify:		1☐Microphallus/ hypospadias 5☐Other:_______<br>2☐Ambiguous genitalia<br>3☐Flank mass<br>4☐Supra pubic mass	
**SECTION 10. INTEGUMENTARY/MUSCULOSKELETAL SYSTEM (IF ABNORMAL, SPECIFY ON THE SIDE)**			
48. Skin texture normal	1☐Yes	2☐No	3☐Don't know

49. Umbilical stump normal	1☐Yes	2☐No	3☐Don't know	
50. Musculoskeletal system normal	1☐Yes	2☐No	3☐Don't know	
**SECTION 11. CENTRAL NERVOUS SYSTEM**				
51. Indicate consciousness status	1☐Conscious	2☐Lethargic	3☐Comatose	
52. Reflexes appropriate for age				
52.1 Moro normal for age	1☐Yes	2☐No	3☐Don't know/not done	
52.2 Sucking normal for age	1☐Yes	2☐No	3☐Don't know/not done	
52.3 Grasp appropriate for age	1☐Yes	2☐No	3☐Don't know/not done	
53. Tone normal	1☐Yes	2☐No	3☐Don't know	
53.1 If abnormal, specify		1☐Increased	2☐Decreased	3☐Flacid
54. Presence of seizures	1☐Absent	2☐Present	3☐Don't know	
55. Quality of cry	1☐Normal	2☐High-pitched	3☐Don't know	
**Section 12. Investigations. Summary of additional investigations ordered at time of admission and subsequently should be entered here. Complete separate form for each investigation done when appropriate.**				
56. **IMAGING.** Any imaging done?	1☐Yes	2 ☐No – Skip to LABORATORY		
57. Chest X-ray	1☐Done	2 ☐Not done		
57.1 If done, findings normal?	1☐Yes	2 ☐No; specify:_______________________________		
58. Abdominal X-ray	1☐Done	2 ☐Not done		
58.1 If done, findings normal?	1☐Yes	2 ☐No; specify:_______________________________		
58.2 NEC diagnosed by x-ray	1☐Yes	1☐No		
59. Other X-ray	1☐Done	2 ☐Not done		
59.1 If done, findings normal?	1☐Yes	2 ☐No; specify:_______________________________		
60. Head ultrasound	1☐Done	2 ☐Not done		

60.1 If done, findings normal?	1☐Yes	2 ☐No; specify:______________________________
60.2 IVH diagnosed by US	1☐Yes	1☐No
61. Abdominal ultrasound	1☐Done	2 ☐Not done
61.1 If done, findings normal?	1☐Yes	2 ☐No; specify:______________________________
62. Other ultrasound<br>SPECIFY:______________________	1☐Done	2 ☐Not done
62.1 If done, findings normal?	1☐Yes	2 ☐No; specify:______________________________
63. MRI	1☐Done	2 ☐Not done
63.1 If done, findings normal?	1☐Yes	2 ☐No; specify:______________________________
**64. LABORATORY** analyses done	1☐Yes	2 ☐No
65. Complete Blood count	1☐Done	2 ☐Not done
65.1 If done, findings normal?	1☐Yes	2 ☐No; specify:______________________________
65.2 If WBC is abnormal,	Specify value	_________________________
65.3 If HB or HCT is abnormal,	Specify value	__________________________
65.4 If platelets is abnormal	Specify value	___________________________
66. Blood gas analysis	1☐Done	2 ☐Not done
66.1 If done, findings normal?	1☐Yes	2 ☐No; specify:______________________________
67. Bilirubin test	1☐Done	2 ☐Not done
67.1 If done, findings normal?	1☐Yes	2 ☐No; specify:______________________________
68. Random blood sugar	1☐Done	2 ☐Not done
68.1 If done, findings normal?	1☐Yes	2 ☐No; specify:______________________________
69. Blood culture	1☐Done	2 ☐Not done

69.1 If done, findings normal?	1☐Yes	2 ☐No; specify:______________________________
70. CSF analysis	1☐Done	2 ☐Not done
70.1 If done, findings normal?	1☐Yes	2 ☐No; specify:______________________________
71. CSF culture	1☐Done	2 ☐Not done
71.1 If done, findings normal?	1☐Yes	2 ☐No; specify:______________________________
72. Other Culture	1☐Done	2 ☐Not done
72.1 if done, location:	1☐Eye	2☐Umbilicus
	3☐Skin	4☐CSF
72.2 If done, findings normal?	1☐Yes	2 ☐No; specify:______________________________
73. Gram stain	1☐Done	2 ☐Not done
73.1 if done, where:	1☐Eye	2☐Umbilicus
	3☐Skin	4☐CSF
73.2 If done, findings normal?	1☐Yes	2 ☐No; specify:______________________________
74. C-reactive protein test?	1☐Done	2 ☐Not done
74.1 If done, findings normal?	1☐Yes	2 ☐No; specify:______________________________
75. ESR done?	1☐Done	2 ☐Not done
75.1 If done, findings normal?	1☐Yes	2 ☐No; specify:______________________________
76. Serum albumin	1☐Done	2 ☐Not done
76.1 If done, findings normal?	1☐Yes	2 ☐No; specify:______________________________
77. VDRL in baby	1☐Done	2 ☐Not done
77.1 If done, findings normal?	1☐Yes	2 ☐No; specify:______________________________
78. HIV test for mother	1☐Done	2 ☐Not done

78.1 If done, findings normal? 1☐Yes 2☐No; specify:______________________________

79. Liver function test (LFT)? 1☐Done 2☐Not done

79.1 If done, findings normal? 1☐Yes 2☐No; specify:_____________________________

80. Serum electrolytes 1☐Done 2☐Not done

80.1 If done, findings normal? 1☐Yes 2☐No; specify:_____________________________

80.2 If abnormal, specify electrolyte: ____

81. Other test? (SPECIFY TEST) 1☐Done 2☐Not done

____

Section 13. Clinical Diagnosis

82. Were any conditions diagnosed? 1☐Yes 2☐No, baby healthy

83. If yes, specify (Tick all that apply):

Respiratory

1☐Respiratory Distress Syndrome
2☐Meconomium aspiration syndrome
3☐Apnea
4☐Pulmonary hemorrhage
5☐Other respiratory; Specify:_________________

Metabolic

6☐Hypoglycemia
7☐Electrolyte disorders
8☐Other metabolic disorders;
SPECIFY_________________

Infection

9☐Early onset neonatal sepsis (<72 hours)
10☐Late onset neonatal sepsis (>=72 hours)
11☐Pneumonia
12☐Meningitis
13☐HIV-exposed
14☐Congenital syphilis
15☐Ophthalmia neonatorum
16☐Other infection; Specify:_________________

Neurologic

25☐ Perinatal asphyxia with multiple organ involvement
26☐Acute bilirubin encephalopathy (ABE)
27☐Seizure disorder
28☐Intraventricular hemorrhage (IVH)
29☐Neural tube defect
30☐Other neurologic; Specify:_________________

Hematologic

17☐ Anemia
18☐ Polycythemia
19☐ DIC
20☐ Rh incompatibility
21☐ ABO incompatibility
22☐ Hyper- bilirubinemia
23☐ Hemorrhagic disease of the newborn
24☐ Other hematologic; Specify:________________

Miscellaneous

31☐ Disseminated intravascular coagulopathy (DIC)
32☐ Feeding problem
33☐ Necrotizing enterocolitis (NEC)
34☐ Congenital malformation
35☐ Chromosomal anomalies
36☐ Congenital heart disease
37☐ Congestive heart failure
38☐ Renal failure
39☐ Traumatic birth injury
40☐ Hypothermia
41☐ Uvelectomy
42☐ Circumcisions (e.g., bleeding, infection)
43☐ *Shock*
44☐ Other; Specify:________________

Section 14: Sequence of clinical events until death (please describe the important changes in illness that led to death)

SECTION 15. FORM COMPLETION

83. Name of person completing this form: ______________________________|___|___|___| (ID)

84. Name of person reviewing this form:_______________________________|___|___|___| (ID)

85. Date of form completion |___|___| -|___|___|-|___|___|___|___| (DD-MM-YYYY)

MITS Gross Specimen Examination Form Study-Specific/Autopsy ID: ____________

Form Instructions: Complete one form per MITS case.

1. Gross Specimen Examination 1
– Samples that should be fixed for a minimum of 4 but no more than 24 hours

Name of person performing gross examination 1	______________________________
Date of Gross Examination 1	_______________ (DD/MM/YYYY)
Time of Gross Examination 1	________ (24 hour)

Sample Name	Marked as collected?	Received in lab?	Sample ID from MITS / lab iD	# Cores/ pieces	Size of smallest and largest core Min	Max	Fragmentation of cores?
**Brain/CNS**	☐ Y ☐ N	☐ Y ☐ N	1_ _ -_ _ _-03-J	_______	______	_____	☐ Y ☐ N
**Left Lung/Thorax**	☐ Y ☐ N	☐ Y ☐ N	1_ _ -_ _ _-05-J1	______	______	_____	☐ Y ☐ N
**Right Lung/Thorax**	☐ Y ☐ N	☐ Y ☐ N	1_ _ -_ _ _-05-J2	______	______	_____	☐ Y ☐ N
**Liver**	☐ Y ☐ N	☐ Y ☐ N	1_ _ -_ _ _-06-J	______	______	_____	☐ Y ☐ N
**Other:** ____________ __	☐ Y ☐ N	☐ Y ☐ N	1_ _ -_ _ _-_ _ -_ _	______	______	_____	☐ Y ☐ N
**Other:** ____________ __	☐ Y ☐ N	☐ Y ☐ N	1_ _ -_ _ _-_ _ -_ _	______	______	_____	☐ Y ☐ N
**Other:** ____________ __	☐ Y ☐ N	☐ Y ☐ N	1_ _ -_ _ _-_ _ -_ _	______	______	_____	☐ Y ☐ N
**Other:** ____________ __	☐ Y ☐ N	☐ Y ☐ N	1_ _ -_ _ _-_ _ -_ _	______	______	_____	☐ Y ☐ N

2. Gross Specimen Examination 2 – Samples that should be fixed for a minimum of 12 hours

Name of person performing gross examination 2	______________________________
Date of Gross Examination 2	_______________ (DD/MM/YYYY)
Time of Gross Examination 2	________ (24 hour)

Sample Name	Marked as collected?	Received in lab?	Sample ID from MITS/ Lab ID	Number of Cores/Pieces present	Size of smallest and largest core Min	Max	Fragmentation of cores?
**Placenta Membrane**	☐ Y ☐ N	☐ Y ☐ N	1_ _ -_ _ _-08-J	_ _	______	____	☐ Y ☐ N
**Umbilical Cord**	☐ Y ☐ N	☐ Y ☐ N	1_ _ -_ _ _-09-J	_ _	______	____	☐ Y ☐ N
**Placenta**	☐ Y ☐ N	☐ Y ☐ N	1_ _ -_ _ _-10-J	_ _	______	____	☐ Y ☐ N
**Other:** ____________	☐ Y ☐ N	☐ Y ☐ N	1_ _ -_ _ _-_ _ -_ _	_ _	______	____	☐ Y ☐ N
**Other:** ____________	☐ Y ☐ N	☐ Y ☐ N	1_ _ -_ _ _-_ _ -_ _	_ _	______	____	☐ Y ☐ N
**Other:** ____________	☐ Y ☐ N	☐ Y ☒ N	1_ _ -_ _ _-_ _ -_ _	_ _	______	____	☐ Y ☐ N

3. Gross Specimen Examination 3
– Samples that should be fixed for a minimum of 24 hours (e.g. bone marrow)

Name of person performing gross examination 3	______________________________
Date of Gross Examination 3	_______________ (DD/MM/YYYY)
Time of Gross Examination 3	________ (24 hour)

Sample Name	Marked as collected	Received in lab?	Sample ID from MITS/ Lab ID	Number of Cores/Pieces present	Size of smallest and largest core Min	Max	Fragmentation of cores?
**Other:** ____________	☐ Y ☐ N	☐ Y ☐ N	1_ _ -_ _ _-_ _ -_ _	_ _	______	____	☐ Y ☐ N
**Other:** ____________	☐ Y ☐ N	☐ Y ☐ N	1_ _ -_ _ _-_ _ -_ _	_ _	______	____	☐ Y ☐ N

PLACE MITS KIT ID HERE

Form Instructions: Complete one form per MITS case, marking positive findings.

1. Preparation

Ensure appropriate personal protective equipment (PPE) is worn by all MITS personnel prior to beginning the MITS procedure: ☐ gown ☐ cap ☐ mask ☐ goggles ☐ shoe covers/boots ☐ gloves (x2)

☐ Confirm MITS kit contains all items in the list of contents, arrange items on disinfected surface, and apply labels to tubes as needed. Gather all materials and reagents not included in the MITS kit prior to starting the MITS specimen collection. Check supplies available in backup box.

☐ **Check name and informed consent.**

2. Overview

Site/Location ____________________ MITS specialist ____________________ MITS assistant ____________________

Time and date of birth __________ (24 hour) ____________________ (DD/MM/YYYY)

Time and date of death __________ (24 hour) ____________________ (DD/MM/YYYY)

Circumstances preventing MITS ☐ Y ☐ N If yes, describe: ____________________

Time and date MITS initiated __________ (24 hour) ____________________ (DD/MM/YYYY)

Placenta submitted? ☐ Y ☐ N ☐ N/A *If yes, complete Placenta Collection Form*

Photographs taken (***check all that apply***): ☐ Front ☐ Back ☐ Side ☐ Nails/Hand ☐ Other gross lesions

☐ No photos taken, explain why: ____________________

If other, describe: ____________________

Is face in full view in the frontal picture? ☐ Y ☐ N

**If additional space is required for description use the "Other notes" in Section 6.*

3. Anthropometric Measurements

Date of measurements ____________________ (DD/MM/YYYY)

Weight ____________________ ☐ g ☐ kg	Height/length ________________ (cm)
MUAC (6m-5y) ____________________ (cm)	Head circumference (up to 12m) ________________ (cm)

Stillbirth/Neonate ONLY:

Right leg length ____________________ (cm) Right foot length ________________ (cm)

4. Body Inspection and Palpation

Date of gross findings ____________________ (DD/MM/YYYY)

Sex ☐ Male ☐ Female ☐ Indeterminate

Evidence of trauma(*check all that apply*): ☐ No ☐ Bruises ☐ Lacerations ☐ Abrasions ☐ Burns ☐ Fractures ☐ Bleeding ☐ Other

If present, describe: ____________________

Appearance of bodyweight ☐ Normal ☐ Obese ☐ Cachectic ☐ Thin

Petechiae ☐ Y ☐ N ☐ No exam If yes, location: ____________________

Rash ☐ Y ☐ N ☐ No exam If yes, location: ____________________

Other skin/mucosal lesions ☐ Y ☐ N If yes, describe: ____________________

Bleeding from(*check all that apply*): ☐ Mouth ☐ Ears ☐ Nose ☐ Rectum ☐ No bleeding ☐ Other

Gross facial abnormalities ☐ Y ☐ N ☐ No exam If yes, describe: ____________________

Jaundice/Icterus/ Cyanosis ☐ Y ☐ N ☐ No exam If yes, describe: ____________________

Abdominal distention ☐ Y ☐ N ☐ No exam

Lymphadenomegaly ☐ Y ☐ N ☐ No exam If yes, location of enlarged lymph nodes: ____________________

Hepatomegaly ☐ Y ☐ N ☐ No exam

Splenomegaly ☐ Y ☐ N ☐ No exam

Umbilicus abnormal ☐ Y ☐ N ☐ No exam If yes, describe: ____________________

Other significant gross findings ☐ Y ☐ N If yes, describe: ____________________

**If additional space is required for description use the "Other notes" in Section 6.*

5. Body Cleaning and Sterilization

Clean the areas of the body to be punctured before beginning the MITS procedure.

Specific areas to be cleaned are indicated in Section 6 for each category of MITS case.

1. Clean with water. Dry with gauze.
2. Clean with abundant alcohol, using circular movements from the center to the periphery. Wait 5 minutes.
3. Clean with abundant iodine, using circular movements from the center to the periphery. Wait 5 minutes.

6. Specimen Collection

Note: Numbers in parentheses indicate the number of cores to be taken

☐ **Stillbirth**<br>Maceration level:<br>☐ None, fresh<br>☐ Grade 1 ☐ Grade 2 ☐ Grade 3	☐ **Neonate**<br>☐ **Infant, open fontanelle**	☐ **Infant, closed fontanelle**<br>☐ **Child**<br>☐ **Adult**
**Cleaning completed:**<br>☐ nuchal area ☐ anterior fontanelle<br>☐ thorax ☐ axillae ☐ abdomen	**Cleaning completed:**<br>☐ nuchal area ☐ anterior fontanelle<br>☐ nares ☐ thorax ☐ axillae<br>☐ abdomen	**Cleaning completed:**<br>☐ nuchal area ☐ nares<br>☐ thorax ☐ axillae ☐ abdomen
**CSF** ☐ Y ☐ N<br>Total volume collected: ____ (ml)<br>☐ Clear ☐ Turbid<br>☐ Purulent ☐ Hematic	**CSF** ☐ Y ☐ N<br>Total volume collected: ____ (ml)<br>☐ Clear ☐ Turbid<br>☐ Purulent ☐ Hematic	**CSF** ☐ Y ☐ N<br>Total volume collected: ____ (ml)<br>☐Clear ☐ Turbid<br>☐ Purulent ☐ Hematic
**CNS, posterior - cryovial** (3)<br>☐ Y ☐ N	**CNS, posterior - cryovial** (3)<br>☐ Y ☐ N	**CNS, posterior - cryovial** (3)<br>☐ Y ☐ N
**CNS, posterior - formalin jar** (6)<br>☐ Y ☐ N	**CNS, posterior - formalin jar** (6)<br>☐ Y ☐ N	**CNS, posterior - formalin jar** (6)<br>☐ Y ☐ N
**CNS, fontanelle - formalin jar** (6)<br>☐ Y ☐ N	**CNS, fontanelle - formalin** jar (6)<br>☐ Y ☐ N	CNS, fontanelle: DO NOT COLLECT
If any of the brain, posterior and fontanelle, specimens were not collected, specify which and why they were not collected: __________	If any of the brain, posterior and fontanelle, specimens were not collected, specify which and why they were not collected: __________	If any of the brain, posterior and fontanelle, specimens were not collected, specify which and why they were not collected: __________
NP swab: DO NOT COLLECT	**NP swab**<br>☐ Y **label tube* ☐ N	**NP swab**<br>☐ Y **label tube* ☐ N
	**NP swab for SARS CoV-2 Testing**<br>☐ Y **label tube* ☐ N	**NP swab for SARS CoV-2 Testing**<br>☐ Y **label tube* ☐ N
CNS transnasal: DO NOT COLLECT	CNS transnasal: DO NOT COLLECT	**CNS, transnasal - formalin jar** (6)<br>☐ Y ☐ N<br>If any of the brain, trans-nasal, specimens were not collected, specify which and why they were not collected: __________
**Blood** ☐ Y<br>Total volume collected: _____ (ml)<br>Location collected from:<br>☐ Supraclavicular ☐ Heart<br>☐ Other: _________<br>Gross appearance of blood:<br>☐ Clear ☐ Turbid<br>☐ Purulent ☐ Hematic<br>1. Culture tube - 0.5-5 ml ☐ Y ☐ N<br>2. EDTA tube - 1.5-9 ml ☐ Y ☐ N<br>**invert EDTA tube after filling*<br>3. Blood spot card circles ☐ Y ☐ N<br>**completely fill circles*	**Blood** ☐ Y ☐ N<br>Total volume collected: _____ (ml)<br>Location collected from:<br>☐ Supraclavicular ☐ Heart<br>☐ Other: _________<br>Gross appearance of blood:<br>☐ Clear ☐ Turbid<br>☐ Purulent ☐ Hematic<br>1. Culture tube - 0.5-5 ml ☐ Y ☐ N<br>2. EDTA tube - 1.5-9 ml ☐ Y ☐ N<br>**invert EDTA tube after filling*<br>3. Blood spot card circles ☐ Y ☐ N<br>**completely fill circles*	**Blood** ☐ Y ☐ N<br>Total volume collected: _____ (ml)<br>Location collected from:<br>☐ Supraclavicular ☐ Heart<br>☐ Other: _________<br>Gross appearance of blood:<br>☐ Clear ☐ Turbid<br>☐ Purulent ☐ Hematic<br>1. Culture tube - 0.5-5ml ☐ Y ☐ N<br>2. EDTA tube - 1.5-9 ml ☐ Y ☐ N<br>**invert EDTA tube after filling*<br>3. Blood spot card circles ☐ Y ☐ N<br>**completely fill circles*

**R & L lung - cryovial** (6) ☐ Y ☐ N<br>If the right and left lung specimen was not collected, specify why it was not collected: __________	**R & L lung - cryovial** (6) ☐ Y ☐ N<br>If the right and left lung specimen was not collected, specify why it was not collected: __________	**R & L lung - cryovial** (6) ☐ Y ☐ N<br>If the right and left lung specimen was not collected, specify why it was not collected: __________
**Left lung - formalin jar** (6) ☐ Y ☐ N<br>If any of the left lung specimens were not collected, specify which and why they were not collected: __________	**Left lung - formalin jar** (6) ☐ Y ☐ N<br>If any of the left lung specimens were not collected, specify which and why they were not collected: __________	**Left lung - formalin jar** (6) ☐ Y ☐ N<br>If any of the left lung specimens were not collected, specify which and why they were not collected:<br>__________
**Right lung - formalin jar** (6) ☐ Y ☐ N<br>If any of the right lung specimens were not collected, specify which and why they were not collected: __________	**Right lung - formalin jar** (6)☐ Y ☐ N<br>If any of the right lung specimens were not collected, specify which and why they were not collected: _______	**Right lung - formalin jar** (6)☐ Y ☐ N<br>If any of the right lung specimens were not collected, specify which and why they were not collected: _______
**R&L Lung/Thorax cryovial SARS CoV-2**<br>☐ Y ☐ N	**R&L Lung/Thorax cryovial SARS CoV-2**<br>☐ Y ☐ N	**R&L Lung/Thorax cryovial SARS CoV-2**<br>☐ Y ☐ N
**Liver - cryovial** (3) ☐ Y ☐ N	**Liver - cryovial** (3) ☐ Y ☐ N	**Liver - cryovial** (3) ☐ Y ☐ N
**Liver - formalin jar** (6) ☐ Y ☐ N	**Liver - formalin jar** (6) ☐ Y ☐ N	**Liver - formalin jar** (6) ☐ Y ☐ N
If any of the liver specimens were not collected, specify which and why they were not collected: __________	If any of the liver specimens were not collected, specify which and why they were not collected: __________	If any of the liver specimens were not collected, specify which and why they were not collected: __________
DO NOT COLLECT	**Rectal swab** ☐ Y ☐ N	**Rectal swab** ☐ Y ☐ N

Additional samples collected (*e.g., skin, effusions, etc.)*

☐ Bone Marrow ☐ Hair ☐ Skin ☐ Urine

*Description*____

Other notes: ____

____

7. Sample Collection Summary

Check all samples taken below.	Histology	Microbiology
CSF	-	☐
Blood	-	☐
Brain/CNS	☐	☐
Left Lung/Thorax	☐	☐
Right Lung/Thorax	☐	☐
Liver	☐	☐
Lung for SARS CoV-2	-	☐
Rectal Swab	-	☐
NP Swab	-	☐
NP Swab for SARS CoV-2	-	☐
Extra 1: ____________________________________	☐	☐
Extra 2: ____________________________________	☐	☐
Other: ____________________________________	☐	☐
Other: ____________________________________	☐	☐

8. End of Procedure

MITS procedure end time: __________ (24 hour)

☐ Check for excessive seepage or bleeding. Clean body with water as needed.

☐ Confirm all the containers and jars are properly labeled and closed.

☐ Confirm any unused, labeled materials either have their labels removed or are disposed of in a biowaste container.

☐ Confirm any unused, unlabeled materials are added to a backup box.

☐ Confirm all sharps are disposed in sharps container.

☐ Confirm MITS rack with all the used cryovials in the MITS cool box.

☐ Confirm MITS tray with the used formalin jars, the unused large screw-cap jar, any tissue cassettes in the MITS kit box.

☐ Confirm all used surfaces are washed and sterilized, digital camera is stored/charging.

MITS Site Pathology Report

PLACE MITS KIT ID HERE

Name of person who completed the form: ________ ________

Date of histological examination: _____ _____ (DD/MM/YYYY)

Form Instructions: Use this form to document gross and histological findings of the tissue specimens.

A. Liver Tissue

1. Autolysis present?	☐ Y ☐ N
2. If yes, describe grade:	☐ Mild ☐ Moderate ☐ Severe
3. Tissue processing inadequate?	☐ Y ☐ N
4. If no, provide reason:	☐ Fixation ☐ Sectioning ☐ Staining
5. Total number of cores present ________	
6. Number of cores with target tissue_________	
7. Other tissues present_________	

B. Liver Tissue Findings

1. Granulomas	☐ No	☐ Mild	☐ Severe
2. Portal inflammation	☐ No	☐ Mild	☐ Severe
3. Sinusoidal inflammation	☐ No	☐ Mild	☐ Severe
4. Pigment in portal macrophages	☐ No	☐ Mild	☐ Severe
5. Pigment in Kupffer cells	☐ No	☐ Mild	☐ Severe
6. Steatosis	☐ No	☐ Mild	☐ Severe
7. Necrosis	☐ No	☐ Mild	☐ Severe
8. Fibrosis	☐ No	☐ Mild	☐ Severe
9. Cholestasis	☐ No	☐ Mild	☐ Severe
10. Congestion	☐ No	☐ Mild	☐ Severe

C. Liver Tissue Diagnosis

1. Liver diagnosis unremarkable	☐ Y	☐ N
2. Extramedullary hematopoiesis	☐ Y	☐ N
3. Steatosis (large droplet, small droplet)	☐ Y	☐ N
4. Sinusoidal leukocytosis	☐ Y	☐ N
5. Sepsis	☐ Y	☐ N
6. Cholestasis	☐ Y	☐ N
7. Viral hepatitis	☐ Y	☐ N
8. Active hepatitis	☐ Y	☐ N
9. Chronic hepatitis	☐ Y	☐ N
10. Neonatal hepatitis	☐ Y	☐ N
11. Granulomas	☐ Y	☐ N
12. Paucity of bile ducts	☐ Y	☐ N
13. Fibrosis	☐ Y	☐ N
14. Past malaria	☐ Y	☐ N
15. Active malaria	☐ Y	☐ N
16. Other diagnosis	☐ Y	☐ N
17. If other diagnosis, please specify: ____________________________		

D. Lesions (Liver)

1. Other lesions in liver? ☐ Y ☐ N
2. If other lesions in liver, please specify: ____
3. Lesions in organs other than liver? ☐ Y ☐ N
4. If yes, please describe other lesions in organs other than liver: ______________________

MITS Site Pathology Report

E. Right Lung Tissue

1. Autolysis present? ☐ Y ☐ N
2. If yes, describe grade: ☐ Mild ☐ Moderate ☐ Severe
3. Tissue processing inadequate? ☐ Y ☐ N
4. If no, provide reason: ☐ Fixation ☐ Sectioning ☐ Staining
5. Total number of cores present __________
6. Number of cores with target tissue_________
7. Other tissues present__________

F. Right Lung Tissue Findings

1. Neutrophilic infiltrate in alveoli	☐ No	☐ Mild	☐ Severe
2. Hyaline membranes	☐ No	☐ Mild	☐ Severe
3. Interstitial inflammation	☐ No	☐ Mild	☐ Severe
4. Viral cytopathic effect	☐ No	☐ Mild	☐ Severe
5. Necrosis	☐ No	☐ Mild	☐ Severe
6. Fungi	☐ No	☐ Mild	☐ Severe
7. Necrotizing granulomas	☐ No	☐ Mild	☐ Severe
8. Non-necrotizing granulomas	☐ No	☐ Mild	☐ Severe
9. Intra-alveolar hemorrhage	☐ No	☐ Mild	☐ Severe
10. Aspirated material	☐ No	☐ Mild	☐ Severe
11. Intra-alveolar proteinaceous material	☐ No	☐ Mild	☐ Severe
12. Intra-alveolar hemosiderosis	☐ No	☐ Mild	☐ Severe
13. Edema	☐ No	☐ Mild	☐ Severe
14. Fibrin	☐ No	☐ Mild	☐ Severe
15. Increased alveolar macrophages	☐ No	☐ Mild	☐ Severe

G. Right Lung Tissue Diagnosis

1. Right lung diagnosis unremarkable	☐ Y	☐ N
2. Bacterial pneumonia	☐ Y	☐ N
3. Interstitial pneumonitis	☐ Y	☐ N
4. Hyaline membrane disease	☐ Y	☐ N
5. Diffuse alveolar damage	☐ Y	☐ N
6. Bronchopneumonia without infectious agents identified	☐ Y	☐ N
7. Viral pneumonia	☐ Y	☐ N
8. Fungal pneumonia	☐ Y	☐ N
9. Aspiration pneumonia	☐ Y	☐ N
10. Granulomas, necrotizing	☐ Y	☐ N
11. Granulomas, non-necrotizing	☐ Y	☐ N
12. Findings compatible with intrauterine fetal stress	☐ Y	☐ N
13. Other diagnosis	☐ Y	☐ N
14. If other diagnosis, please specify: ______________		

H. Lesions (Right Lung)

1. Other lung lesions? ☐ Y ☐ N
2. If other lesions in the lung, please specify: ____
3. Lesions in organs other than the target tissue (right lung)? ☐ Y ☐ N
4. If yes, please other lesions in organs other than the right lung: _______________________

I. Left Thoracic Organs, Left lung and Heart

1. Total number of cores present: _____ 2. Other tissues present:___

J. Left Lung Tissue

1. Number of cores with target tissue__________

2. Autolysis present?	☐ Y ☐ N
3. If yes, describe grade:	☐ Mild ☐ Moderate ☐ Severe
4. Tissue processing inadequate?	☐ Y ☐ N
5. If no, provide reason:	☐ Fixation ☐ Sectioning ☐ Staining

K. Left Lung Tissue Findings

1. Neutrophilic infiltrate in alveoli	☐ No ☐ Mild ☐ Severe
2. Hyaline membranes	☐ No ☐ Mild ☐ Severe
3. Interstitial inflammation	☐ No ☐ Mild ☐ Severe
4. Viral cytopathic effect	☐ No ☐ Mild ☐ Severe
5. Necrosis	☐ No ☐ Mild ☐ Severe
6. Fungi	☐ No ☐ Mild ☐ Severe
7. Necrotizing granulomas	☐ No ☐ Mild ☐ Severe
8. Non-necrotizing granulomas	☐ No ☐ Mild ☐ Severe
9. Intra-alveolar hemorrhage	☐ No ☐ Mild ☐ Severe
10. Aspirated material	☐ No ☐ Mild ☐ Severe
11. Intra-alveolar proteinaceous material	☐ No ☐ Mild ☐ Severe
12. Intra-alveolar hemosiderosis	☐ No ☐ Mild ☐ Severe
13. Edema	☐ No ☐ Mild ☐ Severe
14. Fibrin	☐ No ☐ Mild ☐ Severe
15. Increased alveolar macrophages	☐ No ☐ Mild ☐ Severe

L. Left Lung Tissue Diagnosis

1. Left lung diagnosis unremarkable	☐ Y ☐ N
2. Bacterial pneumonia	☐ Y ☐ N
3. Interstitial pneumonitis	☐ Y ☐ N
4. Hyaline membrane disease	☐ Y ☐ N
5. Diffuse alveolar damage	☐ Y ☐ N
6. Bronchopneumonia without infectious agents identified	☐ Y ☐ N
7. Viral pneumonia	☐ Y ☐ N
8. Fungal pneumonia	☐ Y ☐ N
9. Aspiration pneumonia	☐ Y ☐ N
10. Granulomas, necrotizing	☐ Y ☐ N
11. Granulomas, non-necrotizing	☐ Y ☐ N
12. Findings compatible with intrauterine fetal stress	☐ Y ☐ N
13. Other diagnosis	☐ Y ☐ N
14. If other diagnosis, please specify: ____________________________________	

M. Lesions (Left Lung)

1. Other lung lesions? ☐ Y ☐ N
2. If other lesions in the lung, please specify: ____
3. Lesions in organs other than the target tissue (right lung)? ☐ Y ☐ N
4. If yes, please other lesions in organs other than the right lung: ____________________

N. CNS Tissue

1. Autolysis present? ☐ Y ☐ N	☐ Y ☐ N
2. If yes, describe grade:	☐ Mild ☐ Moderate ☐ Severe
3. Tissue processing inadequate?	☐ Y ☐ N
4. If no, provide reason:	☐ Fixation ☐ Sectioning ☐ Staining

5. Total number of cores present: _________
6. Number of cores with target tissue:_________
7. Other tissues present:_______

O. CNS Tissue Findings		
1. Meninges present	☐ Y	☐ N
2. Grey matter present	☐ Y	☐ N
3. White matter present	☐ Y	☐ N

P. CNS Tissue Findings Continued			
1. Meningitis, neutrophilic	☐ No	☐ Mild	☐ Severe
2. Meningitis, lymphocytic	☐ No	☐ Mild	☐ Severe
3. Meningoencephalitis	☐ No	☐ Mild	☐ Severe
4. Encephalitis	☐ No	☐ Mild	☐ Severe
5. Viral cytopathic effect	☐ No	☐ Mild	☐ Severe
6. Cerebral malaria	☐ No	☐ Mild	☐ Severe
7. Fungi	☐ No	☐ Mild	☐ Severe
8. Necrotizing granulomas	☐ No	☐ Mild	☐ Severe
9. Non-necrotizing granulomas	☐ No	☐ Mild	☐ Severe
10. Necrosis	☐ No	☐ Mild	☐ Severe
11. Gliosis	☐ No	☐ Mild	☐ Severe
12. Glial nodules	☐ No	☐ Mild	☐ Severe
**13. Intraventricular hemorrhage**	☐ No	☐ Mild	☐ Severe

Q. CNS Tissue Diagnosis		
1. CNS diagnosis unremarkable	☐ Y	☐ N
2. Neutrophilic meningitis	☐ Y	☐ N
3. Lymphocytic meningitis	☐ Y	☐ N
4. Meningoencephalitis	☐ Y	☐ N
5. Perivascular inflammation	☐ Y	☐ N
6. Viral encephalitis	☐ Y	☐ N
7. Granulomas, necrotizing	☐ Y	☐ N
8. Granulomas, non-necrotizing	☐ Y	☐ N
9. Fungal lesions	☐ Y	☐ N
10. Granulomas	☐ Y	☐ N
11. Vasculitus, thrombosis	☐ Y	☐ N
**12. Intraventricular hemorrhage**	☐ Y	☐ N
13. Other CNS diagnosis	☐ Y	☐ N
14. If other CNS diagnosis, please specify: ______________________________________		

R. Lesions (CNS)		
1. Other CNS lesions?	☐ Y	☐ N
2. If other CNS lesions, please specify: ____		
3. Lesions in organs other than the target tissue (CNS)?	☐ Y	☐ N
4. If yes, please describe other lesions in organs other than the CNS: _____________		

S. Central Nervous System (Trans-nasal)			
1. Autolysis present?	☐ Y	☐ N	
2. If yes, describe grade:	☐ Mild	☐ Moderate	☐ Severe
3. Tissue processing inadequate?	☐ Y	☐ N	
4. If no, provide reason:	☐ Fixation	☐ Sectioning	☐ Staining
5. Total number of cores present _ ________			
6. Number of cores with target tissue________			
7. Other tissues present________			

T. CNS (Trans-nasal) Tissue Findings

1. Meninges present	☐ Y	☐ N
2. Grey matter present	☐ Y	☐ N
3. White matter present	☐ Y	☐ N
4. Nasal content present	☐ Y	☐ N

U. CNS (Trans-nasal) Tissue Diagnosis		
1. CNS diagnosis unremarkable	☐ Y	☐ N
2. Neutrophilic meningitis	☐ Y	☐ N
3. Lymphocytic meningitis	☐ Y	☐ N
4. Meningoencephalitis	☐ Y	☐ N
5. Perivascular inflammation	☐ Y	☐ N
6. Viral encephalitis	☐ Y	☐ N
7. Granulomas, necrotizing	☐ Y	☐ N
8. Granulomas, non-necrotizing	☐ Y	☐ N
9. Fungal lesions	☐ Y	☐ N
10. Granulomas	☐ Y	☐ N
11. Vasculitus, thrombosis	☐ Y	☐ N
**12. Intraventricular hemorrhage**	☐ Y	☐ N
12. Other CNS diagnosis	☐ Y	☐ N
13. If other CNS diagnosis, please specify: ______________________________________		

V. CNS (Trans-nasal) Tissue Findings Continued			
**1.**Meningitis present	☐ No	☐ Mild	☐ Severe
**2.**Grey matter present	☐ No	☐ Mild	☐ Severe
3.White matter present	☐ No	☐ Mild	☐ Severe
4. Nasal content present	☐ No	☐ Mild	☐ Severe
5. Meningitis, neutrophilic	☐ No	☐ Mild	☐ Severe
6. Meningitis, lymphocytic	☐ No	☐ Mild	☐ Severe
7. Meningoencephalitis	☐ No	☐ Mild	☐ Severe
8. Encephalitis	☐ No	☐ Mild	☐ Severe
9. Viral cytopathic effect	☐ No	☐ Mild	☐ Severe
10. Cerebral malaria	☐ No	☐ Mild	☐ Severe
11. Fungi	☐ No	☐ Mild	☐ Severe
12.. Necrotizing granulomas	☐ No	☐ Mild	☐ Severe
13.. Non-necrotizing granulomas	☐ No	☐ Mild	☐ Severe
14. Necrosis	☐ No	☐ Mild	☐ Severe
15. Gliosis	☐ No	☐ Mild	☐ Severe
16. Glial nodules	☐ No	☐ Mild	☐ Severe
17. Intraventricular hemorrhage	☐ No	☐ Mild	☐ Severe

W. Lesions (CNS Trans-nasal)		
1. Other CNS lesions?	☐ Y	☐ N
2. If other CNS lesions, please specify: ____		
3. Lesions in organs other than the target tissue (CNS)?	☐ Y	☐ N
4. If yes, please describe other lesions in organs other than the CNS: ____________		

X. Placenta and Cord			
1. Autolysis present?	☐ Y	☐ N	
2. If yes, describe grade:	☐ Mild	☐ Moderate	☐ Severe
3. Tissue processing inadequate?	☐ Y	☐ N	
4. If no, provide reason:	☐ Fixation	☐ Sectioning	☐ Staining
Placenta: ☐ Y ☐ N	Cord:	☐ Y	☐ N

Y. Placenta and Cord Tissue Findings	
1. Chorioamnionitis	☐ No ☐ Mild ☐ Severe
2. Lymphocytic infiltrate	☐ No ☐ Mild ☐ Severe
3. Histiocytic infiltrate	☐ No ☐ Mild ☐ Severe
4. Viral cytopathic effect	☐ No ☐ Mild ☐ Severe
5. Active malaria	☐ No ☐ Mild ☐ Severe
6. Past malaria	☐ No ☐ Mild ☐ Severe
7. Infarction	☐ No ☐ Mild ☐ Severe
8. Thrombi	☐ No ☐ Mild ☐ Severe
9. Fibrosis	☐ No ☐ Mild ☐ Severe
10. Calcification	☐ No ☐ Mild ☐ Severe

Z. CNS (Trans-nasal) Tissue Diagnosis	
1. Placenta/cord diagnosis unremarkable	☐ Y ☐ N
2. Chorioamnionitis	☐ Y ☐ N
3. Infarction	☐ Y ☐ N
4. Villitis	☐ Y ☐ N
5. Active malaria	☐ Y ☐ N
6. Past malaria	☐ Y ☐ N
7. Other diagnosis	☐ Y ☐ N
8. If other CNS diagnosis, please specify: ______________________________________	

Placenta Collection Form

PLACE Placental ID HERE

Study-Specific/Autopsy ID: ______________________________

Form Instructions: Mark only what is present

1. Overview

☐ Following delivery, the cord and placenta should be collected.

☐ Samples for PCR/Microbiology are collected prior to placement in formalin and as soon as possible.

☐ Weight and photographs should be taken PRIOR to fixation of the placenta in formalin for > 24 hours.

Site ID ____________________ MITS specialist ______________________ MITS assistant ______________________

Date of exam ____________________(DD/MM/YYYY) Time of exam __________________ (24 hour)

2. Placental Disc and Description

Note: Measurements should be taken prior to fixation

Photographs taken (check all that apply): ☐ Maternal side ☐ Fetal side ☐ Cord and membranes ☐ Other gross lesions

Is the fetal side normal: ☐ Y ☐ N - If No, Describe:____

Is the maternal side normal: ☐ Y ☐ N - If No, Describe:____

Weight (*cord and membranes removed*) _________________ (g)

Maximal linear length _________________ (cm)

Maximal linear width _________________ (cm)

Pregnancy was: ☐ Singleton ☐ Twin ☐ Triplet ☐ Other

Presence of hematoma: ☐ Y ☐ N If yes, Specify:____

If hematoma:

% of maternal surface involved ____________ (%)

Subchorionic fibrin: ☐ Y ☐ N If yes, Specify:____

Masses: ☐ Y ☐ N If yes, Specify:____

Other gross findings: ☐ Y ☐ N

If yes, Describe:____

3. Umbilical Cord

Diameter of umbilical cord _________________ (cm)

Length of umbilical cord _________________ (cm)

Distance between insertion and nearest placental margin _________________ (cm)

Velamentous insertion of umbilical cord: ☐ Y ☐ N If yes, Specify:____

Presence of knots: ☐ Y ☐ N If yes, Specify:____

Appearance of umbilical cord: ☐ Hypercoiled ☐ Hypocoiled ☐ Other, Specify:____

Presence of thrombosis: ☐ Y ☐ N If yes, Specify:____

Presence of strictures: ☐ Y ☐ N If yes, Specify:____

Presence of discoloration: ☐ Y ☐ N If yes, Specify:____

Number of vessels: ☐ 2 ☐ 3 If yes, Specify:____

4. Membranes

Describe color: ☐ White ☐ Brown/Green ☐ Red/purpose ☐ Other, Specify:________________________________

Describe opacity: ☐ Clear ☐ Slightly opaque ☐ Opaque ☐ Other, Specify:________________________________

Are membranes complete: ☐ Y ☐ N ☐ Other (specify): ________________________________

Are the membranes:

☐ Circumvallate: ________% ☐ Circummarginate__________%

☐ Neither ☐ Other, Specify:________________________________

☐ Make membrane roll

Placenta Collection Form

5. Placenta Fixation and Sectioning

☐ Fix placenta for 24 hours. *There should be a 10:1 ratio formalin to placenta*

☐ Following fixation rinse placenta with water to remove formalin

☐ Section

1. "Breadloaf" entire placenta at 1-2 cm intervals
2. Photograph the sections together (*all on the same tray)*
3. Re-evaluate fixed specimen for lesions (*infarcts may be more apparent after fixation*)

Mural minimal thickness ________________ (cm)

Mural maximal thickness ________________ (cm)

Any additional lesions: ☐ Y (If yes, photograph) ☐ N ☐ Other, Specify:____________________

If lesions: Total number____________

Location ☐ Central/paracentral ☐ Peripheral ☐ Diffuse

☐ Other, Specify:____________________

Increased parenchymal fibrin: ☐ Y ☐ N If Yes, Specify:____________________

Subchorionic fibrin: ☐ Y ☐ N If Yes, Specify:____________________

Masses: ☐ Y ☐ N If Yes, Specify:____________________

Calcification: ☐ Y ☐ N If Yes, Specify:____________________

Presence of infarcts: ☐ Y ☐ N If Yes, Specify:____________________

If infarcts: Total number____________

% of total parenchymal volume____________ (%)

Presence of hematoma: ☐ Y ☐ N ☐ Other, Specify:____________________

If hematoma: ☐ Marginal ☐ Retroplacental ☐ Subchorionic

% of maternal surface involved ____________ (%) ☐ N/A

Other gross findings: ☐ Y ☐ N

If yes, Describe:____________________

6. Placental Sample Collection

Check all samples taken below.

SAMPLE	SAMPLE ID
Extra placental membrane roll	☐
Umbilical cord - 5cm fetal end	☐
Umbilical cord - 5cm from placental insertion	☐
Full thickness normal appearing placenta - 1	☐
Full thickness normal appearing placenta - 2	☐
Disc membrane junction	☐
Placental lesion 1 (Describe)	☐
Placental lesion 2 (Describe)	☐
Placental lesion 3 (Describe)	☐

7. End of Procedure

☐ Confirm all the containers and jars are properly labeled and closed.

☐ Confirm any unused cryovials and jars are disposed of in a biowaste container.

☐ Confirm all used surfaces are washed, and the digital camera is stored and charging.

STILLBIRTH EVALUATION FORM		FORM_07
MITS SURVEILLANCE ALLIANCE	MITS KIT ID Here: ______________________________<br>Study-Specific/Autopsy ID: ________________________	Version 1.1<br>Page **1 of 1**

This form should be completed by the clinician immediately after delivery of all stillbirths.

SECTION A. INFANT IDENTIFICATION

1. Date of birth: |__|__|-|__|__|-|__|__|__|__| (DD – MM – YYYY)
2. Time of birth (time in 24 hours/min): |__|__|-|__|__|(HH-MM)
3. Infant Sex 1 ☐ Female 2 ☐ Male 3 ☐ DK
4. Was fetus known to be dead prior to delivery? 1 ☐ YES 2 ☐ NO 3 ☐ DK
5. How long before delivery did the mother feel fetal movement?
   |__|__| (DD) |__|__| (HH) 1 ☐ DK

SECTION B. COMPLICATIONS

1. Meconium present? 1 ☐ YES 2 ☐ NO 3 ☐ DK
2. Traumatic Lesions? 1 ☐ YES 2 ☐ NO 3 ☐ DK
   a. If YES -> 1 ☐ Bruising 2 ☐ Fractures
      3 ☐ Lacerations 4 ☐ Fragmentation/dismemberment
      5 ☐ Other specify_______________________________________
3. Cord Prolapse? 1 ☐ YES 2 ☐ NO 3 ☐ DK
4. Nuchal Cord? 1 ☐ YES 2 ☐ NO 3 ☐ DK

SECTION C. ANTHROPMETRIC MEASUREMENTS

1. Birth weight (in grams): |__|__|__|__| g
2. Birth length (crown -heel) (in centimeters): |__|__|. |__|cm
3. Head circumference: |__|__|.|__|cm
4. Foot (toe-heel) length: |__|__|.|__|cm

SECTION D. MACERATION AND FRAGMENTATION AND ABNORMALITIES

1. Was fetus fragmented? 1 ☐ YES 2 ☐ NO 3 ☐ DK
2. Signs of maceration? 1 ☐ YES (Grade I-V) 2 ☐ NO 3 ☐ DK
3.

SECTION E. GENERAL APPERANCE AND EXAMINATION

IF ANY ABNORMAL FINDING SPECIFY DETAILS IN F

1. Structural abnormality present?
   1 ☐ YES 2 ☐ NO (SKIP to Q13.) 3 ☐ DK
2. Head 1 ☐ Normal 2 ☐ Anencephaly 3 ☐ Encephalocele
   4 ☐ Unusual shape 5 ☐ Hydrocephaly 6 ☐ DK
   7 ☐ Other, Specify_________________________________

3.	Face	1 ☐ Normal	2 ☐ Abnormal	3 ☐ DK
4.	Eye	1 ☐ Normal	2 ☐ Abnormal	3 ☐ DK
5.	Nose	1 ☐ Normal	2 ☐ Abnormal	3 ☐ DK
6.	Ears	1 ☐ Normal	2 ☐ Abnormal	3 ☐ DK
7.	Mouth	1 ☐ Normal	2 ☐ Abnormal	3 ☐ DK
8.	Neck	1 ☐ Normal	2 ☐ Abnormal	3 ☐ DK
9.	Chest	1 ☐ Normal	2 ☐ Abnormal	3 ☐ DK

10. Abdomen 1 ☐ Normal 2 ☐ Abdominal wall defect
    3 ☐ DK 4 ☐ Other abnormal, Specify________
11. Spine 1 ☐ Normal 2 ☐ Neuro tube defect
    3 ☐ DK 4 ☐ Other abnormal, Specify________

12.	Skin	1 ☐ Normal	2 ☐ Abnormal	3 ☐ DK
13.	Signs of Trisomy	1 ☐ YES	2 ☐ NO	3 ☐ DK
14.	Photograph taken	1 ☐ YES	2 ☐ NO	3 ☐ DK
15.	Consent for MITS	1 ☐ YES	2 ☐ NO	3 ☐ DK
16.	Consent for Autopsy	1 ☐ YES	2 ☐ NO	3 ☐ DK

SECTION F. SPECIFY ANY ABNORMAL OR OTHER FINDINGS

1. Specify____
   ____
   ____
   ____
   ____

Name of person who completed the form: _________________

Date of completion: __________ (DD/MM/YYYY)
